# Detection of EWS/FLI-1 by Immunostaining. An Adjunctive Tool in Diagnosis of Ewing's Sarcoma and Primitive Neuroectodermal Tumour on Cytological Samples and Paraffin-Embedded Archival Material

**DOI:** 10.1080/13577149977839

**Published:** 1999-03

**Authors:** Gunnar Nilsson, Min Wang, Johan Wejde, Andris Kreicbergs, Olle Larsson

**Affiliations:** 1 Department of Cellular and Molecular Tumour Pathology Cancer Center Karolinska Karolinska Hospital Stockholm S-17176 Sweden; 2 Department of Orthopaedics Karolinska Hospital Stockholm S-17176 Sweden

## Abstract

*Purpose.* Recently we showed that the 68-kDa fusion protein derived from the *EWS/FLI1* hybrid gene can be specifically detected by Western blotting using a polyclonal antibody to the C-terminal of FLI1 on biopsy material from Ewing's sarcoma. The aim of this study was to investigate whether this antibody also could be used for immunocytochemistry and immunohistochemistry in diagnosis of Ewing's sarcoma.

*Methods.* Immunostaining on paraffin-embedded archival material, fine-needle aspirates and tumour touch imprints from Ewing's sarcomas and primitive neuroectodermal tumours (PNET) for detection of the fusion protein was performed. Most cases were also analysed by Western blotting.Tumours of differential diagnostic importance were also included.

Results. Eighty per cent (12/15 cases) of the Ewing tumours exhibited a positive immunoreactivity for the FLI1 antibody. The signal was mainly localised in the nuclei of the tumour cells, which seems reasonable since *EWS/FLI1* is a transcription factor. The signal was found to be specific since it did not appear when the blocking peptide was added to the antibody solution.Moreover, two other types of small-round cell tumours (i.e. neuroblastoma and alveolar rhabdomyosarcoma) were negative as well as most normal tissues.

*Discussion.* Immunostaining of histological and cytological specimens with the FLI1 antibody can be of diagnostic relevance in Ewing tumours carrying t(11;22).The absence of immunoreactivity in non-Ewing cells is most likely due to a low expression of the wild-type FLI1 protein.


*Sarcoma* (1999) **3**, 25–32

ORIGINAL ARTICLE

### Detection of EWS/FLI-1 by immunostaining. An adjunctive tool in diagnosis of Ewing's sarcoma and primitive neuroectodermal tumour on cytological samples and paraffin-embedded archival material

GUNNAR NILSSON[1,2], MIN WANG[1], JOHAN WEJDE[1], ANDRIS KREICBERGS[2], & OLLE LARSSON[1]

[1]*Department of Cellular and Molecular Tumour Pathology, Cancer Center Karolinska, Karolinska Hospital, S-17176 Stockholm, Sweden &* [2]*Department of Orthopaedics, Karolinska Hospital, S-17176 Stockholm, Sweden.*

**Abstract**

*Purpose.* Recently we showed that the 68-kDa fusion protein derived from the *EWS/FLI1* hybrid gene can be specifically detected by Western blotting using a polyclonal antibody to the C-terminal of FLI1 on biopsy material from Ewing's sarcoma. The aim of this study was to investigate whether this antibody also could be used for immunocytochemistry and immunohistochemistry in diagnosis of Ewing's sarcoma.

*Methods.* Immunostaining on paraffin-embedded archival material, fine-needle aspirates and tumour touch imprints from Ewing's sarcomas and primitive neuroectodermal tumours (PNET) for detection of the fusion protein was performed. Most cases were also analysed by Western blotting. Tumours of differential diagnostic importance were also included.

*Results.* Eighty per cent (12/15 cases) of the Ewing tumours exhibited a positive immunoreactivity for the FLI1 antibody. The signal was mainly localised in the nuclei of the tumour cells, which seems reasonable since *EWS/FLI1* is a transcription factor. The signal was found to be specific since it did not appear when the blocking peptide was added to the antibody solution. Moreover, two other types of small-round cell tumours (i.e. neuroblastoma and alveolar rhabdomyosarcoma) were negative as well as most normal tissues.

*Discussion.* Immunostaining of histological and cytological specimens with the FLI1 antibody can be of diagnostic relevance in Ewing tumours carrying t(11;22). The absence of immunoreactivity in non-Ewing cells is most likely due to a low expression of the wild-type FLI1 protein.

**Key words**: *immunocytochemistry, immunohistochemistry, cytology, fusion protein, FLI1, Ewing tumour.*

### Introduction

Morphological diagnosis of Ewing's sarcoma and primitive neuroectodermal tumours (PNET), based on biopsies or fine-needle aspirates, is often associated with severe difficulties.[1–3] Ewing tumours (ETs) are recognised as tumour tissue composed of small round cells, and can be misdiagnosed as other small-cell tumours like lymphoma, neuroblastoma and rhabdomyosarcoma, or even benign conditions like osteomyelitis.[4–7] ETs are seen mainly in childhood and in this age group it accounts for approximately one fourth of all malignancies, or 29 per million children.[5] Since ET is very primitive, no structural, enzymatic or cell surface characteristics specific for this entity exist. Diagnosis is therefore frequently made by excluding other differential diagnostic possibilities.[6] Over the last years several ET-specific translocations have been discovered. Ninety per cent of the ET cases carries the translocation t(11;22)(q24;q12), 5% t(21;22)(q22;q12) and <1% t(7;22)(p22;q12).[8–12] Recently, two additional chromosomal translocations in Ewing's sarcoma have been described, t(17;22) and t(2;22).[13,14] Analysis of translocations by reverse transcription—polymerase chain reaction (RT-PCR) is a useful option in the diagnosis of ET.[15] A limitation, however, with RT-PCR in surgical pathology is the requirement for a strict and rapid handling of fresh material to avoid degradation of tumour cell mRNA. The possibility of simply analysing the product of the *EWS/FLI1* fusion gene [i.e. t(11;22)] on a processed surgical specimen would therefore provide an important diagnostic tool, especially if suitable material for RT-PCR is not available. Recently, we applied Western blotting, using an antibody against the carboxy terminal of the FLI1 protein (sc-356 or C-19), for detection of the 68-kDa *EWS/FLI1* fusion protein in surgical biopsies of Ewing's sarcoma. We could confirm that

Correspondence to: Olle Larsson, Department of Cellular and Molecular Tumour Pathology, Cancer Center Karolinska, Karolinska Hospital, S-17176 Stockholm, Sweden. Tel: +46-8-51775021; Fax: +46-8-7588397; E-mail: olle.larsson@onkpat.ki.se

1357-714X/99/010025-08 $9.00 © 1999 Taylor & Francis Ltd this antibody is highly specific since the fusion protein was only detected in Ewing's sarcoma cells carrying t(11;22)(q24;q12).[16] The lowest detection level for total protein was 0.3 μg.

Antibodies against the MIC2 gene product are commonly used in diagnosis of Ewing's sarcoma.[17,18] However, several other tumours (including lymphoma and rhabdomyosarcoma) have been reported to be immunoreactive to MIC-2 antibodies.[18,19] In the present study we have investigated whether the FLI1 antibody can be used for detection of EWS/FLI1 using immunocytochemistry and immunohistochemistry. Such an application would be very useful in diagnosis of ET on cytological and paraffin-embedded samples.

### Methods

### *Chemicals*

Sc-356 (C-19) (Santa Cruz Biotechnology, Santa Cruz, USA) is a rabbit polyclonal IgG antibody raised against a peptide corresponding to amino acids 434–452 mapping at the carboxy terminus of the FLI1 protein. The epitope is localised closer to the *C*-terminus compared to the ETS binding domain, which is essential for the binding of the transcription protein to DNA.[20,21] A blocking peptide (sc-356 P) (Santa Cruz Biotechnology) was used to confirm the specificity of the FLI-1 antibody. The secondary antibody used for Western blotting was goat anti-rabbit IgG-HRP (sc-2004) (Santa Cruz Biotechnology) and for immunocytochemistry and immunohistochemistry we used a biotinylated anti-rabbit IgG (BA-1000) (Vector Laboratories, Burlingame, USA). The MIC2 gene product was detected by CD99 (DAKO, Glostrup, Denmark). All other chemicals, unless not stated otherwise, were from Sigma Chemicals, St Louis, MO, USA.

### *Cell lines*

The Ewing's sarcoma cell line HTB-166 carrying the t(11;22) *(EWS/FLI1)* translocation, the breast cancer cell line MDA 231, the human colonic carcinoma cell line WiDr, and the human melanoma cell line SK-MEL-2 were obtained from American Type Culture Collection, USA. TTC-466, carrying the t(21,22)(q22;q12) *(EWS/ERG)* translocation, was kindly provided by Dr. P. Sorensen (Department of Pathology, BC Research Institute for Child and Family Health, Vancouver, Canada). Simian virus-40 transformed human fibroblasts (line 90VAVI) were from Dr. G. Stein (Department of Molecular, Cellular and Developmental Biology, University of Colorado, Boulder, CO, USA). The synovial sarcoma cell line A-2243 was kindly provided by Dr. S.A. Aaronson (Mt. Sinai Medical Center, New York, USA). The human diploid fibroblasts (line GM 08333) were obtained from Coriell Institute of Medical Research, NJ, USA and cultured in minimum essential medium supplemented with 10% (v/v) fetal calf serum, 2 mM L-glutamine, 1 mM sodium pyruvate, 1× non-essential amino acids, 0.15 mg/ml benzyl-penicillin and 0.15 mg/ml streptomycin. The cell culture conditions and media for the other cell lines have been described elsewhere.[16]

### *Tumour material*

Fresh-frozen surgical biopsies from clinical cases were used for Western blotting and/or immunocytochemistry. Two fine-needle aspirates and nine tumour touch imprints were put on Superfrost slides (Menzel-Glaser, Germany) and air-dried for immunostaining. Sections for immunostainings were obtained from formalin-fixed paraffin-embedded archival material.

### *Immunohistochemistry and immunocytochemistry*

Immunostaining was performed using the standard ABC-technique (Vector, Elite Standard Kit. cat. PK-6100). Paraffin sections were deparaffinised, rehydrated and subjected to microwaves (700 W) for 5 min. The endogenous peroxidase activity of the pre-treated sections and cytological slides was blocked by hydrogen peroxide ($H_2O_2$) dissolved in methanol (3% $H_2O_2$ : methanol, 1:5 by volume) for 30 min. Sections and slides were then rinsed and incubated with blocking serum (normal horse serum) for 20 min. Excess serum was drained and the slides were incubated with the Sc-356 (C-19) antibody at a 1:200 dilution or CD99 at 1:300 dilution. Sc-356 was incubated overnight at +8°C and CD99 was incubated for 1 h at room temperature. A biotinylated antirabbit IgG was used as a secondary antibody and followed by the ABC-complex. The peroxidase reaction was developed using DAB (Diaminobenzidine tetrahydrochloride, 0.6 mg/ml with 0.03% $H_2O_2$) for 6 min. Counterstaining was performed. Tris-phosphate buffered saline (pH 7.6) was used for rinsing between the different steps.

### *Protein isolation*

Total protein was isolated as described elsewhere.[22] Tumour samples were washed twice with phosphate-buffered saline (PBS) and weighed. Half of each tumour sample was homogenised in a buffer containing 0.32 M sucrose, 1 mM taurodeoxycholic acid, 2 mM $MgCl_2$, 1 mM EDTA, 25 mM benzamidine, 1 μg/ml bacitracin, 2 mM phenylmethylsulphonyl fluoride, 10 μg/ml aprotinin, 10 μg/ml soybean trypsin inhibitor and 10 μg/ml leupeptin. After a 10-min centrifugation at 600×g at 4°C the pellet, containing unbroken cells and cytoskeleton, was discarded. The supernatant was used for analysis. The concentration of total protein was measured using the Bio-Rad protein assay (Bio-Rad, Germany) according to the method of Bradford.[23]

*Gel electrophoresis*

Proteins were dissolved in a sample buffer containing 0.0625 M Tris–HCl (pH 6.8), 20% glycerol, 2% sodium dodecyl sulphate (SDS), bromophenol blue and 100-mM dithiothreitol. Samples of variable protein concentrations were analysed by sodium dodecyl sulphate–polyacrylamide gel electrophoresis (SDS–PAGE) with a 4% stacking gel and a 10% separation gel at 100 V overnight, essentially according to the protocol of Laemmli.[24] The 5× running buffer was prepared by mixing 15 g Tris base, 72 g glycine and 5 g SDS in 1 l distilled water, pH 8.3. A 20 μl SeeBlue Pre-Stained Standard (NOVEX, San Diego, USA) was run simultaneously. Three gel-elecrophoreses were run for each material, one of which was stained with Coomassie blue to monitor the quality of the proteins. The two other gels were used for Western blotting.

*Western blotting*

After SDS–PAGE the separated proteins were transferred at 100 V for 3 h to a Hybond-ECL nitrocellulose membrane (Amersham Life Science, Buckinghamshire, UK). The transfer buffer was prepared by mixing 12.15 g Tris base, 56.25 g glycine and 1 l methanol in 5 l $dH_2O$. The membranes were subsequently blocked for 1 h at room temperature with a blocking solution containing 10% (w/v) skimmed milk powder and 0.3% (v/v) Tween 20 in PBS, pH 7.5. The membranes were incubated with the primary antibody C-19 for 1 h with 10% skimmed milk and 0.3% Tween in PBS. The primary antibody dilution was 1:500. After two washings, the membranes were incubated with goat anti-rabbit IgG-HRP at a 1:500 dilution for 1 h. After washings the membranes were incubated in ECL Western blotting detection reagents (Amersham) for 1 min. The membranes were exposed to Hyperfilm-ECL for 1 min, 5 min or overnight, whereupon detection was performed.

**Results**

Fresh-frozen biopsy material from a typical case of ET was used in the first experiment. Separate samples were used for Western blotting and for immunostaining of fine-needle aspirates. Western blotting using the FLI1 antibody showed a 68-kDa product corresponding to the EWS/FLI1 fusion protein (Fig. 1a). As a control, a sample from normal fibroblasts was also analysed. This sample was found to be negative (Fig 1a). It was confirmed in a separate experiment that the 68-kDa band did not appear if the control peptide (sc-356P) was added to the antibody solution (data not shown). Figure 1(b) shows immunostaining with sc-356 (C-19) of the fine-needle aspirate of the same case. There was a clear positive immunoreactivity of the cells, and this was mainly confined to the cell nuclei. In Fig. 1(c) the sc-356 immunoreactivity in a fine-needle aspirate of another typical ET case is shown.

In Fig. 2 it is demonstrated that a formalin-fixed

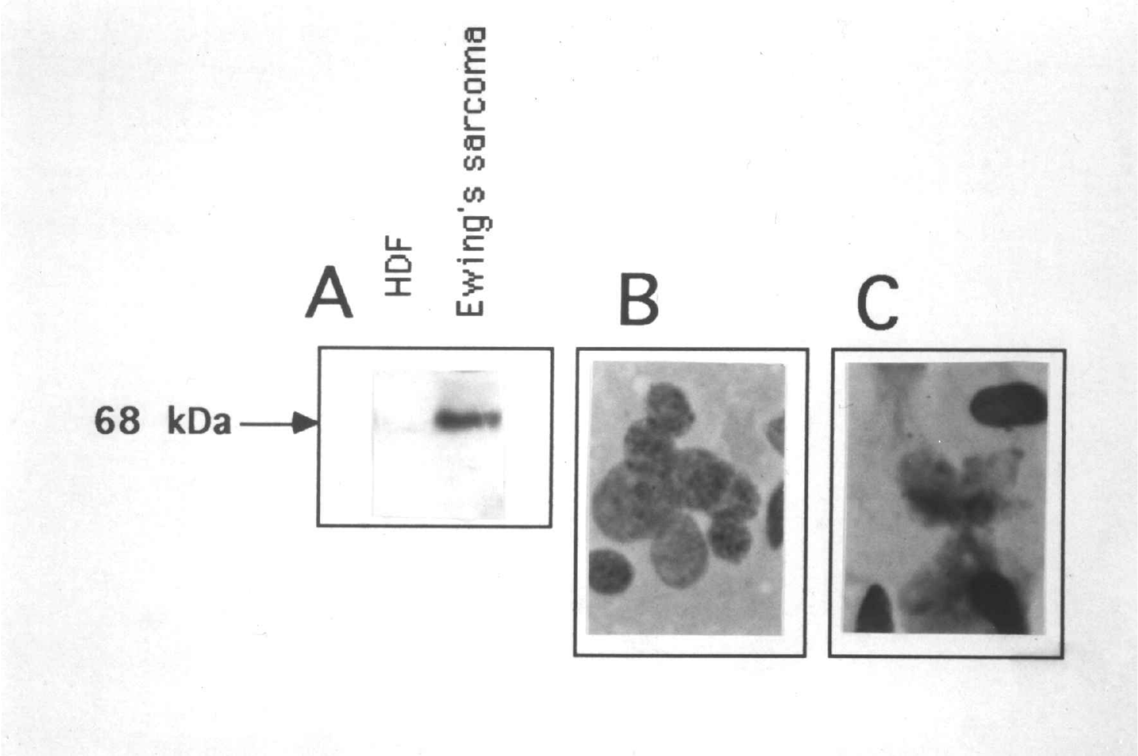

**Fig. 1.** *(a) Detection by Western blotting of the 68-kDa EWS/FLI1 fusion protein in a ET sample using the FLI1 antibody sc-356 (C-19). (b) Immunostaining with the FLI1 antibody on a fine-needle aspirate material from the aforementioned ET sample. (c) Immunostaining with the FLI1 antibody on cytological sample from another ET case. (×400)*

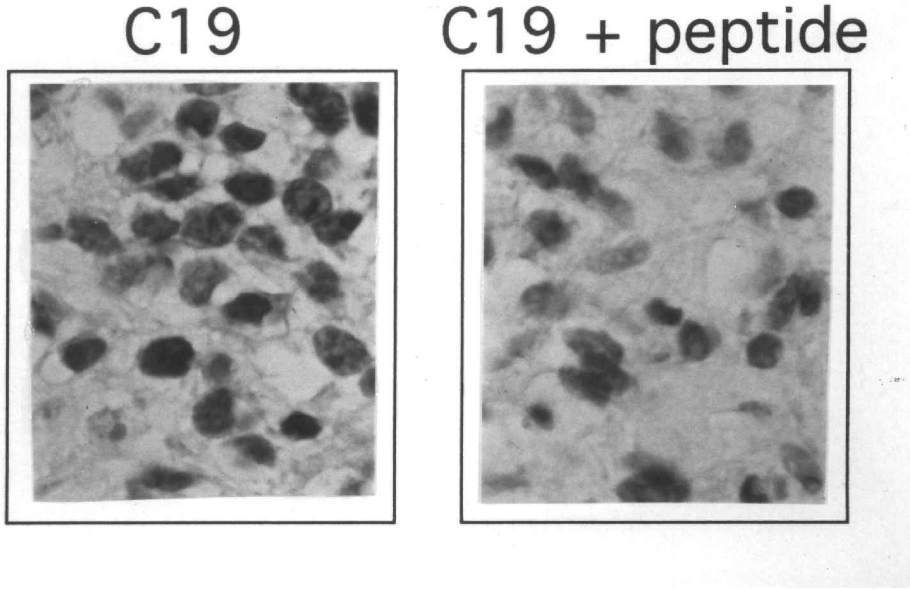

**Fig. 2.** *Immunohistochemistry using the FLI1 antibody sc-356 (C-19) on a paraffin-embedded ET specimen (left panel). Specificity on the FLI1 antibody was tested using the peptide against which the FLI1 antibody was raised (right panel). The peptide was added at concentration exceeding the antibody concentration ten-fold. (×400)*

and paraffin-embedded archival specimen of ET was positively stained by sc-356 (C-19). Even in this case the immunoreactivity was mainly confined to the nuclei. In order to get this positive signal, antigen retrieval using microwave treatment was necessary (see Materials and Methods). As can be seen in the right panel of Fig. 2, the immunoreactivity was totally lost if the control peptide (sc-356P) was added together with the primary antibody during the staining procedure. In a separate experiment we could confirm that sc-356P did not decrease the immunoreactivity of irrelevant antibodies (like MIC-2) (data not shown). Taken together, these results strongly suggest that sc-356 (C-19) does not cross-react with other proteins in the cells.

Figure 3 shows the result from Western blotting using the sc-356 (C-19) antibody on various types of ET and non-ET cells and tissues. The purpose of this experiment was to investigate if there was any detectable wild-type FLI1 in the cells. A positive immunostaining for FLI1 in the non-ET cells would considerably decrease the utility of immunostaining

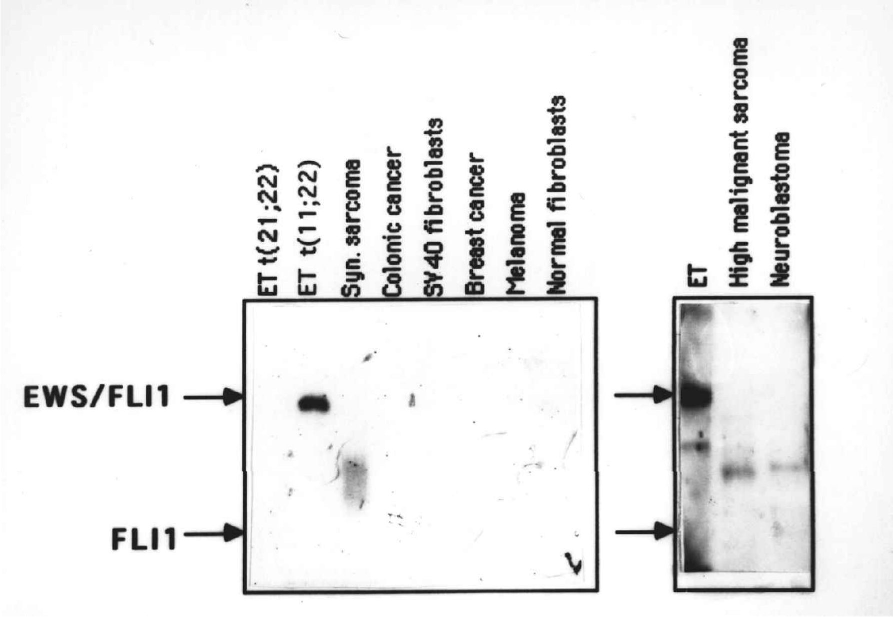

**Fig. 3.** *Analysis for FLI1 and EWS/FLI1 expression by Western blotting in various cell types and tissues. ET t(11;22) represented by HTB-166 cells, ET t(21;22) by TTC-466 cells, syn. sarcoma by A-2243 cells, colonic cancer by WiDr cells, SV40-transformed fibroblast by 90VAVI cells, breast cancer by MDA 231 cells, melanoma by SK-MEL-2 cells, and normal fibroblasts by GM 08333. Upper arrow indicates 68 kDa and lower indicates 51 kDa. High malignant sarcoma refers to malignant fibrous histiocytoma.*

in distinguishing between ET and non-ET cells. As demonstrated all samples analysed, including ET cells with the t(21;22) translocation and neuroblastoma tissue, showed no positive signals for FLI1, which has a molecular weight of 51 kDa.

In Fig. 4(a–d) immunochemistry of four different cases of ET (with known t(11;22) translocations), and in Fig. 4(e–f) immunohistochemistry of two cases of small-round cell non-ET tumours (i.e. neuroblastoma and alveolar rhabdomyosarcoma), are shown. All ET cases were positively stained, whereas the non-ET cases showed no significant immunoreactivity. Two additional cases of rhabdomyosarcoma and neuroblastoma were found to be negative for the FLI1 antibody (data not shown). Two cases of non-Hodgkin's lymphoma were also negatively stained

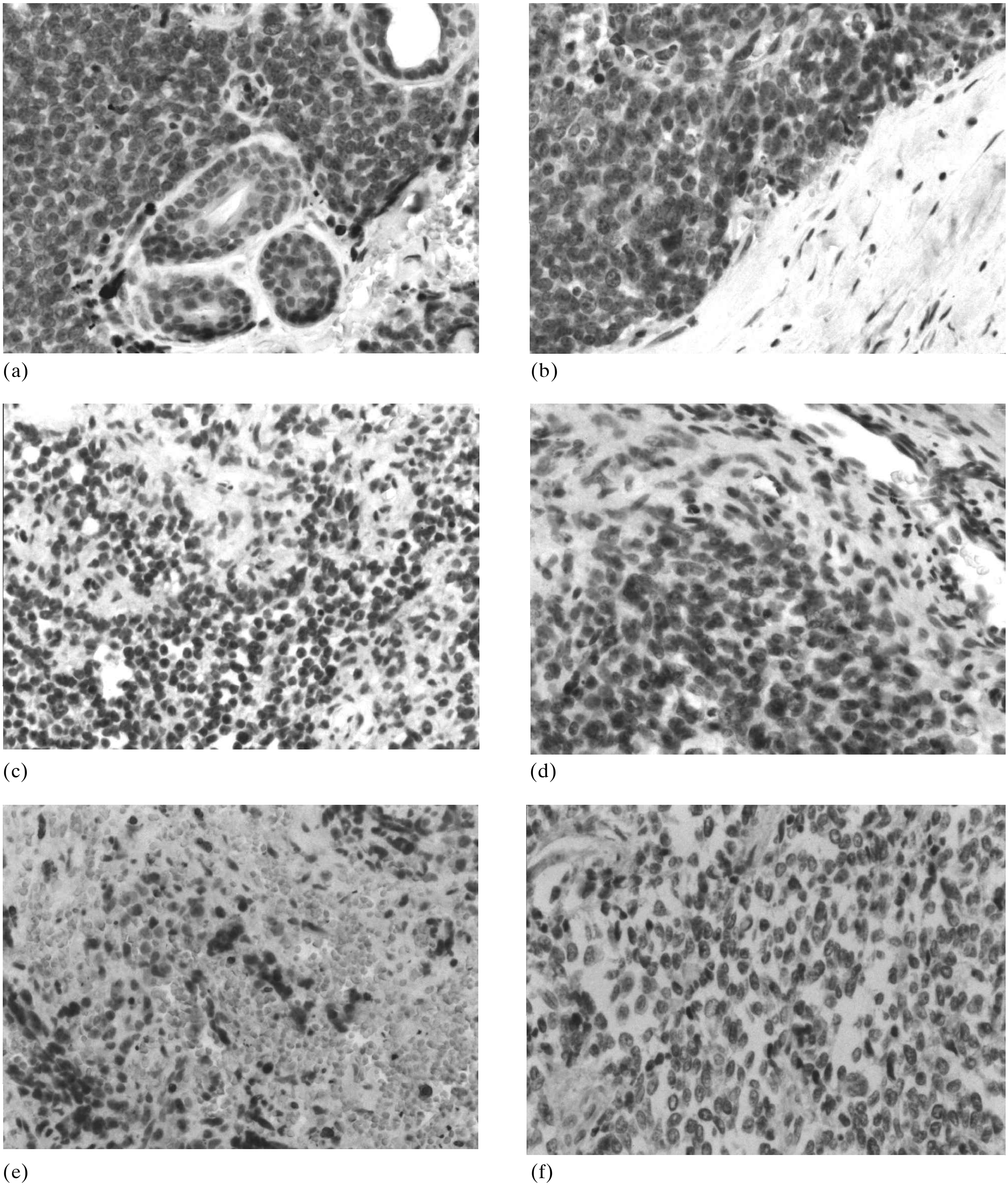

**Fig. 4.** *Immunohistochemistry using the FLI1 antibody on four different paraffin-embedded ET specimen (a–d), as well as on a neuroblastoma (e), and an alveolar rhabdomyosarcoma (f). (×400)*

**Table 1**. *Immunoreactivity of MIC-2 and EWS/FLI-1 in nine additional ET cases**

| Case | Mic-2 (CD-99) | Sc-356 (C-19) | Western blotting (EWS/FLI-1) |
|---|---|---|---|
| 1 | ND† | ND | + |
| 2 | + | – | + |
| 3 | + | + | + |
| 4 | + | + | + |
| 5 | + | + | + |
| 6 | + | – | – |
| 7 | + | + | + |
| 8 | ND | ND | + |
| 9 | + | + | + |

*Slides with tumour touch imprints were subjected to immunostaining with sc-356 and CD99 (see Materials and Methods). Cases containing positively stained cells were then determined microscopically. Two of the cases could not, for technical reasons, be evaluated by immunocytochemistry.

†Not detectable for technical reasons.

(Table 2 below). However, Western blotting analysis of two other lymphoma cases (out of four analysed cases) showed positive signals for wild-type FLI1 (Table 2).

To further evaluate the FLI1 antibody we prepared tumour touch imprints from nine other fresh frozen ET biopsies. Immunostaining was performed with sc-356 (C-19) and CD-99 (MIC-2). The results are presented in Table 1. Parallel biopsy samples were analysed for 68-kDa fusion protein using Western blotting. Unfortunately, the quality of these samples was not good enough for RT-PCR due to RNA degradation. However, the isolated proteins were confirmed to be intact as assayed by SDS–PAGE and Coomassie Blue staining. Therefore we could compare the immunostainings with Western blotting data. Eight cases showed a positive 68-kDa signal for the fusion protein in the Western blotting analyses, five of which were also positive in the immunostainings. Two other immunoimprints could not be evaluated by technical reasons. The case being negative in Western blotting (case 6) was also negative for C-19 in the immunoimprints (Table 1). This case was, however, positive for MIC-2. The immunonegativity of case 6 could be due to an alternative translocation, e.g. t(2;22), t(7;22), t(17;22) or t(21;22). In only one of the cases there was a discrepancy between Western blotting and immunostaining (case 2) (Table 1). This might be explained by that the EWS/FLI-1 expression was too low to be detected by immunocytochemistry. We also tested the immunoreactivity in different types of normal human tissues, including bone, muscle and skin. Apart from an intermediate immunoreactivity in endothelial cells of some vessels, all other tissues investigated were negative (Table 2). In some cases inflammatory cells adjacent to infiltrates to Ewing tumour tissue showed a slightly positive immunostaining (Table 1). However, in four cases of

**Table 2**. *Immunoreactivity of C-19 in some tumour and normal tissues**

| Tissue | Immunoreactivity† |
|---|---|
| Ewing's sarcoma (positive control) | 3+ |
| Lymphoma | 0‡ |
| Inflammatory cells adjacent to ET | 1+ |
| Osteomyelitis | 0 |
| Skeletal muscle | 0 |
| Smooth muscle | 0 |
| Bone | 0 |
| Connective tissue | 0 |
| Vessels | 0–2+§ |
| Lung | 0 |
| Liver | 0 |
| Brain | 0 |
| Kidney | 0 |

*One section of formalin-fixed paraffin-embedded material for each tissue was subjected to immunostaining with C-19 (see Material and Methods). The intensity of nuclear immunoreactivity was then scored.

†Arbitrary scale 0–3+ is based on the intensity of immunoreactivity.

‡Two cases showed no immunoreactivity in immunohistochemical slides. Two out of four cases showed a clear signal for wild type FLI-1 in Western blotting.

§Some vessels showed positive staining in the endothelial cells.

osteomyelitis the inflammatory cells were negatively stained (Table 2).

### Discussion

The molecular analysis of the t(11;22) rearrangements is likely to be of diagnostic value in Ewing's sarcoma and PNET.[25] Moreover, molecular analysis of tumour-associated gene rearrangements comprises an important tool in disclosing mechanisms of oncogenesis. It seems clear that the formation of a transcription factor from the *EWS/FLI1* hybrid gene is a necessary step in tumourigenesis of ET.[26] Recently, we showed that the 68-kDa *EWS/FLI1* fusion protein can be detected by Western blotting using an antibody (C-19) against the carboxy terminal of the FLI1 protein.[16]

The aim of this study was to investigate whether immunostaining with C-19 could be used in diagnosis of ET. Using another FLI1-specific antibody, Melot *et al.* could detect the *EWS/FLI1* fusion protein by immunofluorescense on cell lines.[27] As shown in the present study, positive immunostaining of ET was found in both cytological samples and formalin-fixed paraffin-embedded surgical specimens. The immunoreactivity was mainly localised in the nuclei of the tumour cells, which seems reasonable since the *EWS/FLI1* protein functions as a transcription factor.[28] It was confirmed that the immunoreactivity was not due to cross-reactivity to other proteins. In order to perform specific diagnosis of ET using immunostaining with C-19 it is of great importance that the wild-type FLI1 protein is not, or only slightly, expressed in normal tissue and in tumours that pose differential diagnostic problem, like neuroblastoma and rhabdomyosarcoma. In this study we could confirm that C-19 immunostainings of neuroblastoma and rhabdomyosarcomas were negative. Furthermore, most normal tissues, with the exception of endothelial cells and inflammatory cells, were not immunoreactive. We also tested various types of non-ET cells and ET cells (including ET carrying t(21;22)) for the wild-type FLI1 protein using Western blotting. All of these were also found to be negative. In contrast, Western blotting analysis of some lymphoma tissues showed a positive signal corresponding to wild type FLI1. This result is not surprising since it is known that heamatopoetic cells can express FLI1.[29] Therefore, despite negative immunoreactivity in two lymphoma and four osteomyelitis cases, we believe that immunostaining with C-19 is not fully reliable to distinguish ET from lymphoma. However, the parallel use of alternative markers, like leukocyte common antigen (LCA), in these cases could be helpful in this matter.

Taken together, our present results suggest that immunostaining with FLI1 antibodies can be valuable in the diagnosis of Ewing's sarcoma and PNET, both on cytological material and surgical biopsies.

### Acknowledgments

The authors gratefully acknowledge the following sources of support: Cancer Society in Stockholm (96:118), Lundberg's Research Foundation in Gothenburg, and the Swedish Cancer Society (2992-B96-07XAC).

### References

1 Cavazzana AO, Ninfo V, Roberts J, Triche TJ. Peripheral neuroepithelioma: a light microscopic, immunocytochemical, and ultrastructural study. *Mod Pathol* 1992; 5:71–8.
2 Rettig WJ, Garin-Chesa P, Huvos AG. Ewing's sarcoma: new approaches to histogenesis and molecular plasticity (editorial comment). *Lab Invest* 1992; 66:133–7.
3 Roessner A, Jörgens H. Round cell tumours of bone. *Pathol Res Pract* 1993; 189:1111–36.
4 Thiele CJ. Pediatric peripheral neuroectodermal tumours, oncogenes and differentiation. *Cancer Invest* 1990; 8:629–39.
5 Triche TJ, Askin FB, Kissane JM. Neuroblastoma, Ewing sarcoma, and the differential diagnosis of small, round, blue cell tumour. In: Feingold M, Bennington JC, eds. *Major problem in pathology, vol. 18*. Philadelphia: Saunders, 1987:145–95.
6 Triche TJ. Neuroblastoma and other childhood neural tumours: a review. *Pediat Pathol* 1990; 10:175–93.
7 Tsokos M, Linnoila RI, Chandra RS, Triche TJ. Neuro-specific enolase in the diagnosis of neuroblastoma and other small, round-cell tumours in children. *Hum Pathol* 1993; 15:575–84.
8 Downing JP, Head DR, Parham DM, *et al.* Detection of the t(11;22)(q24;q12) translocation of Ewing's sarcoma and peripheral neuroectodermal tumour by reverse transcription polymerase chain reaction. *Am J Pathol* 1993; 143:1294–300.
9 Turc-Carel C, Aurias A, Mugneret F, *et al.* Chromosomes in Ewing's sarcoma. I. An evaluation of 85 cases of remarkable consistency of t(11;22)(q24;q12). *Cancer Genet Cytogenet* 1988; 32:229–38.
10 Zucman J, Delattre O, Desmaze C, *et al.* Cloning and characterization of the Ewing's sarcoma and peripheral neuroepithelioma t(11;22) translocation break points. *Genes Chromosomes Cancer* 1992; 5:271–7.
11 Sorensen PHB, Lessnick SL, Lopez-Terrada D, Liu XF, Triche TJ, Denny CT. A second Ewing's sarcoma translocation, t(21;22) fuses the EWS gene to another ETS-family transcription factor, ERG. *Nat Genet* 1994; 6:146–51.
12 Jeon I-S, David JN, Braun BS, *et al.* A variant Ewing's sarcoma translocation t(7;22) fuses the Ewing gene to the ETS gene ETV1. *Oncogene* 1995; 10:1229–34.
13 Desmaze C, Brizard F, Turc-Carel C, Melot T, Delattre O, Thomas G, Aurias A. Multiple chromosomal mechanisms generate an EWS/FLI1 or an EWS/ERG fusion gene in Ewing tumors. *Cancer Genet Cytogenet* 1997; 97:12–9.
14 Peter M, Couturier J, Pacquement H, *et al.* A new member of the ETS family fused to EWS in Ewing tumors. *Oncogene* 1997; 14:1159–64.
15 Delattre O, Zucman J, Melot T, *et al.* The Ewing family of tumors—a subgroup of small-round-cell tumors defined by specific chimeric transcripts. *N Engl J Med* 1994; 331:294–9.
16 Wang M, Nilsson G, Carlberg M, *et al.* Specific and sensitive detection of the EWS/FLI1 fusion protein in Ewing's sarcoma by Western blotting. *Virchows Arch* 1998; 432:131–4.

17 Perlman EJ, Dickman PS, Askin FB, Grier HE, Miser JS, Link MP. Ewing's sarcoma—routine diagnostic utilization of MIC2 analysis: a Pediatric Oncology Group/Children's Cancer Group Intergroup Study. *Hum Pathol* 1994; 25:304–7.

18 Weidner N, Tjoe J. Immunohistochemical profile of monoclonal antibody O13: antibody that recognizes glycoprotein p30/32$^{MIC2}$ and is useful in diagnosing Ewing's sarcoma and peripheral neuroepithelioma. *Am J Surg Pathol* 1994; 18:486–94.

19 Mierau GW, Berry PJ, Malott RL, Weeks DA. Appraisal of the comparative utility of immunohistochemistry and electron microscopy in the diagnosis of childhood round cell tumors. *Ultrastruct Pathol* 1996; 20:507–17.

20 Wasylyk B, Hahn SL, Giovane A. The Ets family of transcription factors. *Eur J Biochem* 1993; 211:7–18.

21 Xin JH, Cowie A, Lachance P, Hassel JA. Molecular cloning and characterization of REA3, a new member of the Ets oncogene family that is differentially expressed in mouse embryonic cells. *Genes Dev* 1992; 6:481–96.

22 Gammeltoft S. Peptide hormone receptors. In: Siddle K, Hutton JC, eds. *Peptide hormone action. A practical approach.* Oxford: Oxford University Press, 1990:1–41.

23 Bradford MM. A rapid and sensitive method for the quantitation of microgram quantities of protein utilizing the principle of protein-dye binding. *Anal Biochem* 1976; 72:248–54.

24 Laemmli UK. Cleavage of structural proteins during the assembly of the head of bacteriophage T4. *Nature* 1970; 227:680–5.

25 Dockhorn-Dworniczak B, Schäfer K-L, Dantcheva R, Blasius S, Winkelmann W. Diagnostic value of the molecular genetic detection of the t(11;22) translocation in Ewing's tumours. *Virchows Arch* 1994; 425:107–12.

26 Kovar H. Progress in the molecular biology of Ewing tumors. *Sarcoma* 1998; 2:3–17.

27 Melot T, Gruel N, Doubeikovski A, Sevenet N, Teillaud JL, Delattre O. Production and characterization of mouse monoclonal antibodies to wild-type and oncogenic FLI-1 proteins. *Hybridoma* 1997; 16:457–64.

28 Takatoshi O, Veena N, Rao E, Reddy SP. EWS/Fli-1 chimeric protein is a transcriptional activator. *Cancer Res* 1993; 53:5859–63.

29 Hromas R, May W, Denny C, *et al.* Human FLI-1 localizes to chromosome 11Q24 and has an aberrant transcript in neuroepithelioma. *Biochim Biophys Acta* 1993; 1172:155–8.
